# Transurethral Bipolar Enucleation vs. Transurethral Monopolar Enucleation of the Prostate for the Treatment of Bladder Outlet Obstruction Due to Benign Prostatic Hyperplasia

**DOI:** 10.5152/tud.2026.25075

**Published:** 2026-04-10

**Authors:** Ahmed G Mohamed, Osama Sayed, Ramy Nageib Masoud, Amr Medhat Massoud, Sherif Abdel Rahman Abdul Hay, Ahmed Abdelbary, Mohamed F Elebiary, Hany Fathy

**Affiliations:** 1 Department of Urology, Beni-Suef University Faculty of Medicine, Beni-Suef, Egypt; 2 Department of Urology, Misr University for Science and Technology Faculty of Medicine, Cairo, Egypt; 3 Department of Urology, Cairo University Faculty of Medicine, Cairo, Egypt; 4 Department of Urology, Alazhar University Faculty of Medicine, Cairo, Egypt

**Keywords:** Benign prostatic hyperplasia, bladder outlet obstruction, transurethral bipolar enucleation, transurethral monopolar enucleation

## Abstract

**Objective:**

To compare the feasibility of bipolar transurethral enucleation (B-TUEP) and monopolar transurethral enucleation (M-TUEP) for the management of benign prostatic obstruction.

**Methods:**

This prospective randomized study involved 160 patients: 80 patients who were subjected to B-TUEP; and 80 patients who were subjected to M-TUEP. Patients were evaluated preoperatively and for 1 year postoperatively.

**Results:**

At the 12-month follow-up, no statistically significant intergroup differences were observed in operative time, resected tissue weight, International Prostate Symptom Score, or maximum urinary flow rate. Postoperative rates of re-catheterization for acute urinary retention, urinary incontinence, and urinary tract infections were also comparable between groups. However, the B-TUEP group exhibited significantly less intraoperative bleeding (*P* < .001). The M-TUEP group experienced significantly longer postoperative catheterization, and hospital stays (*P* < .001). Furthermore, the M-TUEP group demonstrated significantly lower mean intraoperative serum sodium levels (*P* < .001), with 1 patient experiencing TUR (Transurethral resection) syndrome.

**Conclusion:**

The findings indicate comparable efficacy between M-TUEP and B-TUEP for benign prostatic hyperplasia treatment, although B-TUEP demonstrates superior safety. Monopolar transurethral enucleation of the prostate remains a safe and effective alternative in the absence of bipolar technology.

**Main Points**

- Comparative feasibility: This prospective randomized study demonstrates that both bipolar (B-TUEP) and monopolar (M-TUEP) transurethral enucleation of the prostate are feasible for large prostates (>80 g), with comparable functional outcomes (International Prostatic Symptom Score (IPSS), Q-max).
- Safety profile: B-TUEP showed a superior safety profile, with significantly less hemoglobin drop (0.98 vs. 1.63 g/dL, *P* < .001), lower risk of TUR syndrome, and shorter catheterization (1.7 vs. 3.8 days) and hospitalization (3.23 vs. 5.94 days) (*P* < .001).
- Operative efficiency: No significant difference in operative time or resected tissue weight between B-TUEP and M-TUEP, though M-TUEP had a higher transfusion rate (2 vs. 1 patient).
- Long-term outcomes: Both techniques achieved similar improvements in voiding parameters (IPSS, Q-max) at 1 week, 1 month, and 3 months, with low rates of complications (e.g., urethral stricture in 1 B-TUEP patient).
- Clinical implications: B-TUEP may be preferred for large prostates due to its safety advantages, while M-TUEP remains a viable alternative with comparable efficacy.

## Introduction

Benign prostatic hyperplasia (BPH) represents the most prevalent urological condition affecting aging men and presents a variable spectrum of lower urinary tract symptoms (LUTS) that significantly impact quality of life.^1^ Transurethral resection of the prostate (TURP) is the established gold standard surgical intervention for managing urinary bladder outlet obstruction (BOO) secondary to BPH.^2^

Although TURP remains the confirmed technique for managing urinary BOO secondary to BPH, it carries significant risks of complications such as bleeding, fluid absorption (TUR syndrome), and prolonged catheter duration.^3^^,^^4^

This has driven the development of alternative procedures, particularly for larger prostates (over 80 g), where safety concerns are amplified. Transurethral enucleation of the prostate (TUEP), often employed as a hybrid procedure (enucleoresection), has emerged as an option to minimize these risks.^5^

Introduced in 1986, monopolar TUEP by Hairoka et al initially lacked widespread adoption owing to its intense learning path and limitations in available instruments.^6^

However, various energy sources have been explored for TUEP. Enucleation of the prostate using holmium laser (HoLEP) has achieved global affirmation and has proven effective through meta-analyses. In 2009, Bach et al integrated the thulium (YAG [Yttrium Aluminum Garnet] laser) into prostate vapoenucleation, leading to the technique now known as thulium laser enucleation of the prostate (Thulium LEP).^7^

Despite the limitations of monopolar electrocautery, particularly regarding TUR syndrome in large prostates, bipolar energy has emerged as an alternative to standard monopolar energy. The high cost of laser equipment has led many surgeons to explore bipolar devices as effective and affordable treatment options for BPH.^8^^,^^9^

The cost of laser platforms used for prostate enucleation remains high, so many surgeons have attempted to perform enucleation using bipolar devices, which are an effective treatment for BPH.^9^ Current surgical literature highlights that the success of endoscopic prostate enucleation hinges primarily on the surgeon’s skill and technique, rather than solely on the energy source employed, thereby renewing interest in monopolar enucleation.^7^ Therefore, this study planned to compare bipolar transurethral enucleation of the prostate (B-TUEP) and monopolar transurethral enucleation of the prostate (M-TUEP) to assess the feasibility of M-TUEP.

## Material and Methods

### Study Design and Ethical Considerations

This prospective, randomized, parallel-group study was conducted at Beni-suef University Hospitals over a 20-month period (March 2021-November 2022). The study protocol was approved by the Institutional Review Board of Faculty of Medicine, Beni-suef University (Ref. No: FMBSUREC/07032021) and adhered to the principles of the Declaration of Helsinki. All participants provided written informed consent prior to enrollment.

### Participant Selection

Eligible participants included men aged >50 years presenting with LUTS secondary to BPH. Inclusion criteria were defined as:

- Prostate volume (PV) ≥80 g (determined by transrectal ultrasound (TRUS)).
- International Prostatic Symptom Score (IPSS) ≥ 8.
- Peak urinary flow rate ≤ Qmax 15 mL/s.

Exclusion criteria encompassed patients with active urinary tract infections (UTI), neurogenic bladder dysfunction, histologically confirmed or clinical suspicion of prostate cancer, previous urethral or prostatic surgery, and severe systemic comorbidities (ASA[American Society of Anesthesiologists] physical status > III).

### Randomization and Blinding

A total of 160 patients were enrolled and randomized in a 1 : 1 ratio using a computer generated randomization list. Patients were allocated to either Group A (Bipolar TUEP, n = 80) or Group B (Monopolar TUEP, n = 80).

### Preoperative Evaluation

A comprehensive preoperative assessment was conducted for all patients, including medical history, physical examination, and digital rectal examination. Laboratory investigations included complete blood count, serum electrolytes (Na+, K+), renal function tests, coagulation profile, and prostate-specific antigen (PSA). Prostate volume was assessed via pelvic and TRUS. Baseline functional status was documented using IPSS and uroflowmetry (Q max).

### Surgical Technique

All procedures were performed under spinal anesthesia by experienced surgeons using a 26-Fr continuous-flow resectoscope.

- Bipolar transurethral enucleation of the prostate group: Enucleation was performed using a bipolar plasma kinetic system (AUTOCON III 400, Karl Storz) with a dedicated enucleating loop (24070 VE-S). Isotonic saline (0.9% NaCl) was utilized for irrigation.
- Monopolar transurethral enucleation of the prostate group: Enucleation was performed using the Erbe VIO 300 D electrosurgical platform and a specialized monopolar loop (27050 CE, Karl Storz) which was compatible with standard monopolar platforms. It was selected due to its mechanical stability during blunt dissection, effective cutting/coagulation control, and reliable handling during the hybrid enucleo-resection technique. Distilled water was used for irrigation.

**Figure 1.**
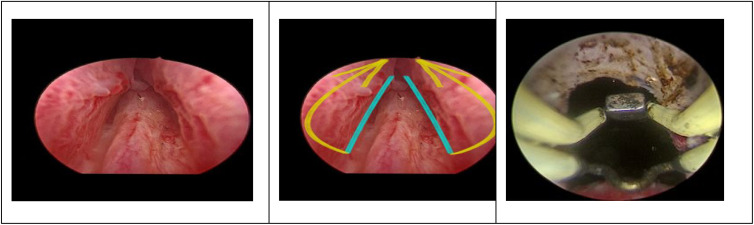
Endoscopic view demonstrates the apical release technique. The blue lines indicate the bilateral mucosal incisions made at the 5 and 7 o’clock positions, extending toward the verumontanum. The yellow arrows depict the trajectory of the circumferential incision progressing anteriorly toward the 12 o’clock position. This dissection separates the mucosa from the external sphincter complex to prevent sphincteric injury during the retrograde displacement of the adenoma toward the bladder neck.

**Figure 2.**
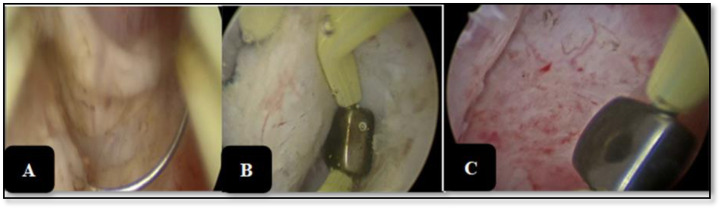
Transurethral enucleation of the prostate technique showing (A): deeping of mucosal incision; (B): left lobe dissected off from the capsule; (C): inner surface of the surgical capsule.

**Figure 3.**
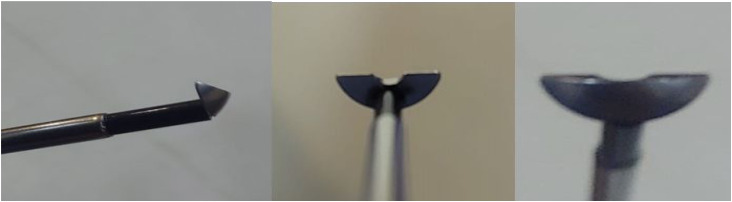
Monopolar enucleation loop by KARL STORZ.

**Figure 4.**
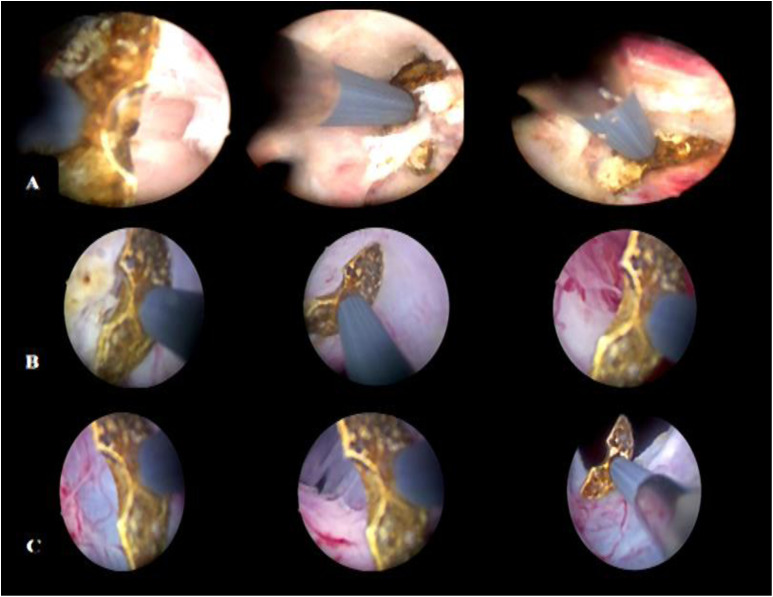
Different steps of monopolar enucleation. (A): Enucleation of the right lobe. (B): Enucleation of the left lobe. (C): Separation of adenoma from the capsule on the left side.

In both cohorts, a “hybrid” enucleo-resection technique was employed. The surgical plane between the adenoma and the surgical capsule was developed using a combination of mechanical blunt dissection and electrosurgical current, mimicking the maneuver of an index finger during open simple prostatectomy. Following complete circumferential mobilization, the devascularized adenoma—remaining attached by a narrow pedicle at the bladder neck—was efficiently respected (Figure 1-4).

### Postoperative Follow-up and Outcomes

,Patients were evaluated at 1 week (post-catheter removal), 1 month, 3 , 6 and 12 months postoperatively. Primary endpoints included changes in IPSS and Q max. Secondary endpoints included perioperative safety parameters (hemoglobin drop, operative time, and TUR syndrome symptoms). Late complications, including urethral stricture and bladder neck contracture, were monitored for up to 12 months.

### Statistical Analysis

Statistical analysis was performed using IBM SPSS Statistics, version 22.0 (IBM Corp., Armonk, NY, USA). Continuous variables are expressed as mean ± standard deviation and were compared using the independent-sample *t*-test (between groups) and paired *t*-test (intra-group longitudinal data). Categorical variables were analyzed using the chi-square X^2^ or Fisher’s exact test. A *P*-value < .05 was considered statistically significant.

## Results

**Figure 5:**
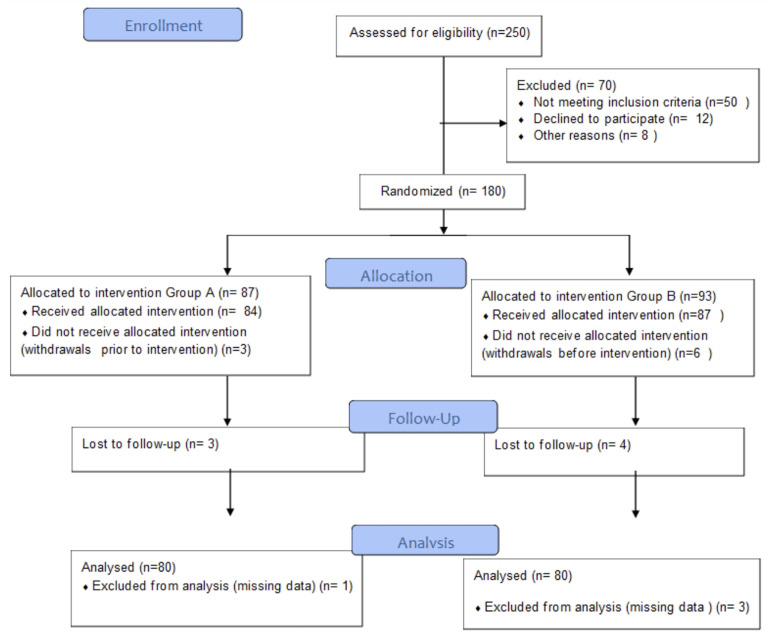
CONSORT (Consolidated Standards of Reporting Trials) flow diagram illustrating the recruitment, randomization, allocation, and follow-up process of the study participants throughout the trial.

A total of 250 patients were assessed for eligibility, of whom 180 were randomized into 2 groups. After excluding patients who did not receive the allocated intervention or were lost to follow-up, 160 patients (80 in each group) completed the study and were included in the final analysis, as detailed in the study flowchart (Figure 5).

The 2 study groups demonstrated comparable preoperative characteristics at baseline, with no statistically significant differences recorded between the groups regarding mean age, PSA level, PV, IPSS, hemoglobin level, or serum sodium level (Table 1).

The mean operative time for M-TUEP (113.56 minutes) was slightly longer than that for B-TUEP (110.43 minutes); however, this was not statistically significant (*P* = .141). Although the mean resected tissue weight was slightly greater in the M-TUEP group (46.17 g) than in the B-TUEP group (45.987 g), this difference was not statistically significant (*P* = .896) (Table 2).

The decrease in the mean hemoglobin concentration was higher in the M-TUEP group (1.63 g/dL) than in the B-TUEP group (0.98 g/dL), with a *P* value of <.001*, the blood transfusion rate was 2 patients in M-TUEB and 1 patient in B-TUEB (Table 2).

The B-TUEP group exhibited significantly greater mean serum sodium levels than did the M-TUEP group (139.83 vs. 138.04 mmol/L, *P* <.001) (Table 2). Notably, 1 patient in the M-TUEP group developed TUR syndrome.

Regarding postoperative catheter duration and hospital stays, the B-TUEP group showed statistically significant shorter catheterization times and shorter hospital stays than the M-TUEP group (1.7 vs. 3.8 days, *P* value <.001) (3.23 vs. 5.94 days, *P* value <.001) (Table 2).

Analysis of IPSS and Q-max improvements following catheter removal showed no statistically significant difference between the B-TUEP and M-TUEP groups in 1 week, 1, 3, , 6and 12 months. The incidence of early urinary incontinence and UTIs remained comparable across both groups and after 12 months. Follow-up data revealed that 1 patient in the B-TUEP group had a urethral stricture requiring endoscopic dilation (Table 3).

## Discussion

Over the past 2 decades, transurethral endoscopic enucleation techniques have increasingly emerged as effective alternatives to conventional TURP, particularly in patients with large prostatic adenomas.^10^ Following the 2016 update of the European Association of Urology guidelines, HoLEP and B-TUEP have been endorsed as first-line surgical options for the management of benign prostatic obstruction in large prostates, owing to their favorable efficacy and safety profiles.^11^

Bipolar transurethral enucleation of the prostate has been established as an effective alternative to HoLEP, utilizing bipolar energy and specially designed enucleation loops to achieve anatomical adenoma dissection. Conversely, M-TUEP, which relies on standard monopolar instruments, represents a cost-effective and technically accessible option, particularly in resource-limited settings. Monopolar transurethral enucleation of the prostate facilitates anatomical enucleation while addressing several limitations associated with TURP, including imprecise plane identification, increased capsular perforation risk, and incomplete adenoma removal.^12^

In the present study, B-TUEP (Group A) and M-TUEP (Group B) were compared for the treatment of LUTS secondary to BPH. The findings demonstrated comparable overall efficacy between the 2 techniques, with no statistically significant differences in operative time, resected tissue weight, postoperative IPSS improvement, or Q-max enhancement. These results support the feasibility of M-TUEP as an effective enucleation technique when bipolar technology is unavailable.

Operative time did not differ significantly between the 2 groups (*P* = .141), indicating comparable procedural efficiency. However, a significantly greater postoperative hemoglobin decline was observed in the M-TUEP group compared with the B-TUEP group (1.63 vs. 0.98 g/dL; *P* < .001). This finding may be attributed to the superior coagulation properties of bipolar energy, which allow more effective sealing of venous sinusoids during enucleation. Although blood transfusion was required in only 3 patients overall, the higher hemoglobin drop in the monopolar group highlights the relative hemostatic advantage of bipolar systems.

Serum sodium changes differed significantly between the 2 groups, with lower postoperative sodium levels observed in the M-TUEP cohort (*P* < .001). One patient in the M-TUEP group developed TUR syndrome, necessitating intensive care admission and medical management. These findings underscore the known limitations of monopolar energy systems, particularly the risk of hypotonic fluid absorption during prolonged procedures. In contrast, bipolar systems utilizing saline irrigation demonstrated improved electrolyte stability.

The results are consistent with those reported by Wang et al^13^ who found no significant difference between B-TUEP and M-TUEP in operative time or hemoglobin decrease, although sodium reduction was more pronounced in the monopolar group. Notably, no cases of TUR syndrome were reported in their series, which may reflect differences in operative duration, irrigation volume, or surgical technique.

With respect to postoperative recovery, catheterization time and hospital stay were significantly shorter in the B-TUEP group. These findings align with the observations of Enikeev et al^7^ who reported reduced catheter duration and length of hospital stay following enucleation procedures. The shorter recovery period associated with B-TUEP may be explained by improved intraoperative hemostasis, reduced postoperative bleeding, and risk of clot retention.

Postoperative complications following catheter removal, including urinary incontinence and UTI were comparable between both groups at all follow-up intervals. Although early transient urinary incontinence was relatively common, its incidence decreased progressively over time, with only a small proportion of patients remaining symptomatic at 3 months. This pattern is consistent with the transient sphincteric dysfunction commonly reported after enucleation procedures. Mourad et al^14^ similarly reported a low incidence of late complications following M-TUEP, including urethral stricture.

The resected tissue weight was comparable between groups, confirming effective adenoma enucleation in both techniques. This finding contrasts with the results of Khalifa Suleiman et al^15^ who reported significantly lower resected tissue volumes in the bipolar group. Such discrepancies may be attributed to variations in surgical experience, prostate morphology, or enucleation technique.

Functional outcomes assessed by IPSS and Q-max demonstrated significant improvement from baseline in both groups, with no statistically significant intergroup differences at any postoperative time point. These results agree with those reported by Proietti et al^16^ who demonstrated substantial early improvements in LUTS and uroflowmetry parameters following M-TUEP, supporting the durability and effectiveness of the monopolar enucleation technique.

## Conclusion

Both B-TUEP and M-TUEP are effective surgical options for the management of benign prostatic obstruction, providing significant and sustained improvement in LUTS and urinary flow parameters. Bipolar transurethral enucleation demonstrated a superior perioperative safety profile, with reduced hemoglobin decline, improved postoperative sodium stability, shorter catheterization time, and shorter hospital stay.

Monopolar transurethral enucleation, while associated with a higher risk of fluid absorption-related complications, remains a safe and effective alternative, particularly in settings where bipolar or laser technologies are unavailable. Its ability to achieve anatomical adenoma enucleation with satisfactory functional outcomes highlights its continued clinical relevance.

Further large-scale, multicenter studies with longer follow-up are warranted to confirm these findings and to establish standardized recommendations for selecting the optimal enucleation modality based on patient characteristics, resource availability, and surgical expertise.

## Figures and Tables

**Table 1.** Preoperative Basic Parameters Between the Studied Group

	**Group A (n = 80)**	**Group B (n = 80)**	***P***
	**Mean ± SD**	**Mean ± SD**	
Age, years	63.5 ± 5.65	62.62 ± 5.84	.334
Total PSA preoperative (ng/mL)	3.64 ± 1.30	3.4 ± 1	.192
prostate volume preoperative (g)	93.6 ± 11.78	91.3 ± 7.92	.148
IPSS score preoperative	23.76 ± 3.963	22.38 ± 6.565	.109
Q-max	10.67 ± 2.836	10.37 ± 2.326	.883
Hemoglobin preoperative (g/dL)	12.975 ± 1.867	12.843 ± 1.256	.557
Sodium level (Na) (mmol/L)	138.78 ± 2.851	138.93 ± 2.828	.783

IPSS, International Prostatic Symptom Score; PSA, prostate-specific antigen.

**Table 2.** Post-Operative Data Operative Time, Weight of Resected Tissue, Hemoglobin Drops and Recovery Between the Studied Groups

	**Group A**	**Group B**	***P***
	**Mean ± SD**	**Mean ± SD**	
Operative time (minutes)	110.43 ± 12.859	113.56 ± 13.945	.141
Weight of resected prostatic tissue (g)	45.987 ± 8.823	46.17 ± 8.682	.896
Hemoglobin drops (g/dL)	0.98 ± 0.43	1.63 ± 0.83	<.001
Sodium serum level (Na) (mmol/L)	139.83 ± 2.416	138.04 ± 3.534	<.001
Catheterization period (day)	1.7 ± 0.726	3.8 ± 0.378	<.001
Hospital stays (day)	3.23 ± 0.634	5.94 ± 0.816	<.001

**Table 3.** Postoperative Data Comparing Q-max, IPSS and Complications

**Items**	**1 week**		***P***	**1 month**		***P***	**3 months**		***P***
	**Group A**	**Group B**		**Group A**	**Group B**		**Group A**	**Group B**	
IPSS (mean ± SD)	12.67 ± 4.36	12.28 ± 4.525	.578	9.25 ± 3.459	9.43 ± 3.173	.731	7.213 ± 1.795	7.13 ± 1.863	.639
Q-max (mean ± SD)	18.37 ± 4.26	17.28 ± 3.965	.095	22.23 ± 3.456	21.73 ± 2.798	.315	22.5 ± 1.60	22 ± 1.13	.774
Urinary incontinence (%)/80	13 (16.3)	16 (20)	.545	8 (10)	11 (13.75)	.464	5 (6.3)	5 (6.3)	1.000
UTI N (%)	8 (10)	10 (12.5)	.617	3 (3.75)	3 (3.75)	1.000	5 (6.3)	4 (5)	.722

No patient requires re-catheterization. Also, 1 patient in B-TUEP showed urethral stricture after 12 months of follow up. At 6 and 12 months of follow-up, both groups maintained significant improvement in IPSS and Qmax compared to baseline, with no statistically significant difference between the two groups.

IPSS, International Prostatic Symptom Score; UTI, urinary tract infection.

## Data Availability

The data that support the findings of this study are available on request from the corresponding author.
